# The Nuclear Ribosomal Transcription Units of Two Echinostomes and Their Taxonomic Implications for the Family Echinostomatidae

**DOI:** 10.3390/biology14081101

**Published:** 2025-08-21

**Authors:** Yu Cao, Ye Li, Zhong-Yan Gao, Bo-Tao Jiang

**Affiliations:** 1 Heilongjiang Academy of Agricultural Sciences Branch of Animal Husbandry and Veterinary Branch, Qiqihar 161005, China; 2 Heilongjiang Provincial Key Laboratory of Veterinary Drugs, Qiqihar 161000, China; 3 Heilongjiang Zhalong National Natural Reserve Administration, Qiqihar 161000, China

**Keywords:** *Echinostoma miyagawai*, *Patagifer bilobus*, ribosomal transcription unit, genetic analysis, phylogenetic analyses

## Abstract

Echinostomatidae is a taxonomically complex group with substantial species diversity and richness. The vast majority of species in this family parasitize birds and mammals, including humans, causing significant economic losses and medical costs. In this study, *Echinostoma miyagawai* (Digenea, Echinostomatidae) and *Patagifer bilobus* (Digenea, Echinostomatidae) were isolated from domestic duck and *Grus japonensis*, respectively. The nearly complete ribosomal transcription unit (rTU) sequences of two echinostomes were obtained, with the rTU for *P*. *bilobus* being obtained for the first time. The nearly complete rTU sequence of *P*. *bilobus* (6790 bp) and *E*. *miyagawai* (6893 bp) encompass the small-subunit (18S) ribosomal DNA (rDNA), internal transcribed spacer 1 (ITS1), 5.8S rDNA, internal transcribed spacer 2 (ITS2), and large-subunit (28S) rDNA. The complete lengths of 18S, ITS1, 5.8S, ITS2, and 28S sequences for *E*. *miyagawai* are 1989 bp, 444 bp, 162 bp, 431 bp, and 3858 bp, respectively. For *P*. *bilobus*, complete or nearly complete lengths of these sequences are 1929 bp (nearly complete), 419 bp, 162 bp, 432 bp, and 3848 bp (nearly complete), respectively. The 18S, ITS, and 28S sequences of *E*. *miyagawai* show the highest sequence similarity with other *E*. *miyagawai*. The ITS and 28S sequences of *P*. *bilobus* show the highest sequence similarity with other *P*. *bilobus*, while 18S sequence shows the highest similarity with *E*. *miyagawai*. This is likely due to the unavailability of the 18S sequence of *P*. *bilobus* in GenBank. Repeat sequences were identified in 18S, ITS1, ITS2, and 28S sequences, with the 28S sequence containing the most repeats and the 5.8S sequence having none. The results of phylogenetic reconstruction indicated that *E*. *miyagawai* clusters with other *Echinostoma* spp., while *P*. *bilobus* clusters with other *Patagifer* spp., forming sister taxa. This study not only provides the first rTU sequence for *P*. *bilobus* but also reinforces the sister group status of *Patagifer* to *Echinostoma* through phylogenetic evidence. Finally, this study represents the first record of the *G*. *japonensis* as a new host for *P*. *bilobus* and the first report of a bird from the crane family (Gruidae) as a host for any echinostome species. These findings are significant as they expand our understanding of the host range and ecological interactions of Echinostomatidae. The data obtained provide a valuable resource of molecular markers for studying the taxonomy, population genetics, and systematics of the family Echinostomatoidea. This research contributes to a more comprehensive understanding of the evolutionary relationships and biodiversity within this complex group of parasites, which is crucial for developing effective strategies to mitigate their impact on both wildlife and human health.

## Simple Summary

This study investigated two Echinostomatidae trematodes, *Echinostoma miyagawai* and *Patagifer bilobus*, taken from domestic duck and *Grus japonensis*, respectively. We sequenced their near-complete ribosomal DNA sequences, obtaining approximately 6.8 kb for each species, and reported the first rDNA sequence for *P*. *bilobus*. Genetic analysis confirmed that each species clusters within its respective genus, supporting the hypothesis that *Patagifer* and *Echinostoma* are sister lineages. *Grus japonensis* is also recorded as a new host for *P*. *bilobus* and the first known crane host for any *Patagifer* species. These findings provide valuable molecular data for future research on the diversity, transmission, and control of this important parasite family.

## 1. Introduction

Echinostomiasis is a neglected parasitic disease caused by intestinal trematodes from the family Echinostomatidae, which includes over 50 genera and more than 350 species [1,2]. Echinostomes mainly inhabit the intestines of waterfowls and mammals, including humans. Additionally, certain echinostome species have also been documented in reptiles and fishes [3]. In the poultry industry, echinostomes have become significant health problems, causing low feed conversion ratios and resulting in economic losses. Moreover, they pose a considerable threat to public health and safety. The life cycle of Echinostomatidae is relatively complex and involves a multi-host indirect life history. The specific process is as follows: Eggs of the adult trematode are excreted from the host’s body via feces. Under suitable environmental conditions, the ovum within the egg begins to divide and develops into a miracidium. After hatching, the miracidium enters the water and swims freely, searching for the first intermediate host (usually aquatic snails). Once the miracidium penetrates the snail, it develops during the sporocyst and redia stages, eventually forming a cercaria. The cercaria can either continue to develop into a metacercaria within the same snail or leave the snail and burrow into other second intermediate hosts (such as other snails, bivalves, fish, frogs, tadpoles, etc.). The metacercaria, which is the infective stage of Echinostomatidae, can be found within the second intermediate host or attached to aquatic plants. Infection occurs when the definitive host consumes the second intermediate host containing infectious metacercariae raw or undercooked [4]. Although echinostomes are distributed worldwide, human echinostomiasis mainly occurs in southeastern, eastern, and northeastern Asian countries due to eating habits [5]. In summary, Echinostomatidae trematodes are significant not only to public health and agriculture but also in ecological research. Understanding their ecological characteristics and practical significance can aid in developing effective control strategies, safeguarding human and animal health, and promoting the sustainable development of ecosystems. A defining feature of echinostomes is the presence of a collar of spines encircling the oral sucker in one or two rows. The quantity and arrangement of these spines are crucial for taxonomic identification. However, the systematic classification of echinostomes remains highly complex and is constantly undergoing revision and phylogenetic analysis [6,7]. Information on the genera, species, intermediate hosts, geographic distribution, and host species of *Echinostoma* spp. and *Patagifer* spp. is listed in Table 1.

The family Echinostomatidae displays a high degree of taxonomic variation, and there has been an important controversy about the taxonomy of echinostomes. Although the number and arrangement of the head spines of echinostomes are key to morphological identification, the count of spines can fluctuate among individuals within the same species or developmental stage, and during the process of preparing specimens, morphological traits and spines might be lost, which can result in misidentification. Molecular techniques utilizing genetic markers have become an indispensable tool in the taxonomy of echinostomes. It is reported that the taxonomy of the species of *Echinostoma* and *Echinoparyphium* can be investigated through the analysis of the mitochondrial DNA (mtDNA) [49,90]. In addition, it is reported that rDNA and mtDNA can be used for the identification of complex species or new species in echinostomes as gene makers [6,62].

Ribosomal DNA is a very important component of the cellular genome, playing a key role in protein synthesis, genome stability, and cell growth and proliferation. The primary function of rDNA is to encode ribosomal RNA (rRNA). Ribosomal RNA is one of the main components of ribosomes and plays a central role in the structure and function of ribosomes. Ribosomal DNA typically exists in the genome as tandem repeats. This repetitive structure can ensure that the cell has sufficient templates to synthesize large amounts of rRNA, thereby meeting the cell’s demand for ribosomes. And the transcriptional unit contains three genes (18S, 5.8S, and 28S rRNA) with two internal transcribed spacers (ITS1 and ITS2) separating the genes and an intergenic spacer (IGS) [91]. Moreover, the rDNA region contains a large number of repetitive sequences, which can act as a buffering mechanism [92]. When the genome is damaged or undergoes mutations, these repetitive sequences can provide additional templates to help repair the damaged genomic regions. Ribosomal DNA has extremely important application value in biological taxonomy, especially in molecular phylogenetics and species identification. Ribosomal DNA in taxonomy has not only improved the accuracy and efficiency of species identification but also provided a powerful tool for studying the evolutionary history of organisms. By analyzing rDNA sequences, scientists can gain a deeper understanding of the phylogenetic relationships among organisms, reveal the kinship between species, and thereby promote the development of biological taxonomy [93]. It is reported rDNA have been crucial in resolving taxonomic issues for trematodes [94,95].

This study isolated two species of Echinostomatidae trematodes, characterized by their plump and elongated leaf-shaped bodies, from the intestines of domestic duck and *G*. *japonensis*, respectively. Based on the morphological characteristics, hosts, and parasitic sites, these two echinostomes were identified as belonging to the genera *Echinostoma* and *Patagifer*. The higher taxa of these two species are listed in Table 2. *Echinostoma* is the type genus of the family Echinostomatidae [4]. The genus Echinostoma can be divided into five groups, one of which, the “revolutum” group, is characterized by 37 “collar-spines” [4,96]. *Echinostoma miyagawai*, a member of the “revolutum” group, primarily parasitizes the intestines of waterfowl, causing symptoms such as diarrhea, anemia, and stunted growth [21,49]. This species has also been documented in humans in China [97,98]. With the family Echinostomatidae, the genus *Patagifer* is relatively less reported. It currently comprises 12 recognized species that mainly parasitize birds of the family Threskiornithidae and are distributed worldwide [88]. In addition to Threskiornithidae birds, *Patagifer* species have also been reported in birds from the families Ardeidae, Podicipedidae, and Scolopacidae [87,99]. However, this study marks the first isolation of a *Patagifer* species from the family Gruidae, thereby enriching the host diversity of *Patagifer* species. The type species of the genus Patagifer, *Patagifer bilobus*, parasitizes the intestines of birds and distributed in Eastern Europe, Egypt, Australia, Brazil, and China [100]. This paper presents the nearly complete rTU sequences of *E. miyagawai* and *P. bilobus*, with the rTU sequence for *P*. *bilobus* being obtained for the first time. The current research aims to study 18S, ITS, and 28S sequences as a genetic marker for the genetic characterization of Echinostomatidae species, the reconstruction of its phylogenetic relationship, and a comparison of these characterizations. The phylogenetic evidence reinforces sister group status of *Patagifer* to *Echinostoma*. These data provide valuable molecular resources for resolving taxonomic ambiguities within the Echinostomatidae family. Lastly, this study represents the first record of *G*. *japonensis* as a new host for *P*. *bilobus*. This finding not only expands our understanding of the host range of *Patagifer* species but also highlights the importance of continued research into the ecological and evolutionary dynamics of Echinostomatidae trematodes. Such research is crucial for developing effective strategies to manage and mitigate the impact of these parasites on both wildlife and human health.

## 2. Materials and Methods

### 2.1. Trematodes Collection and Species Identifcation

*Echinostoma miyagawai* were collected from the intestine of naturally infected domestic duck in Qiqihar City, China. *Patagifer bilobus* were collected from the intestine of naturally infected *G. japonensis* in the Zhalong National Nature Reserve, China, in compliance with the Wildlife Protection Law of the People’s Republic of China (a draft of an animal protection law in China released on 2018). The trematodes were thoroughly washed in physiological saline solution. Preliminary species identification was based on size and host predilection [50,87]. Further species identification was also performed by PCR amplification of the 28S sequence, using the primers reported in a previous study [101].

### 2.2. Primers and Amplifcation

Total genomic DNA was extracted from each sample using the TIANamp Genomic DNA Kit (Tiangen, Beijing, China), following the manufacturer’s instructions. The extracted DNA samples were tested for nucleic acid concentration and stored at −20 °C for subsequent genetic analysis. Five pairs of primers were designed based on the multiple alignments of echinostome rTU sequences, and the primer sequences are listed in Table S1. The PCR reactions (25 µL) were performed using 18.30 µL of distilled water, 2.50 µL of 10 × Ex Taq buffer, 2 µL of dNTP Mixture (2.5 mM), 0.50 µL of each primer (25 mM), 1 µL of extracted DNA, and 0.20 µL of Ex Taq DNA polymerase (5 U/µL). The DNA template of positive control used fluke DNA stored in the laboratory, while the negative control used distilled water. The PCR cycling conditions were as follows: 94 °C for 5 min (initial denaturation), followed by 35 cycles at 94 °C for 30 s (denaturation), 50–65 °C for 1 min (annealing), and 72 °C for 1 min 30 s (extension), with a final extension at 72 °C for 10 min. The PCR products with positive bands were sequenced using the Sanger method (Sangon Biotech (Shanghai) Co., Ltd., Shanghai, China).

### 2.3. Sequence Analysis and Annotation of the Ribosomal Transcription Units

The sequences were assembled manually and aligned against the Echinostomatidae trematode sequences in GenBank to identify gene boundaries, using the program DNAStar v. 5.0 [102]. The edited sequences were submitted to GenBank for accession ID numbers. The AT and GC content were calculated using DNAStar v. 5.0 [102]. Forward, reverse, complement, and palindromic repeats were examined by REPuter [103]. These repeats were ≥10 bp with a maximum computed repeats of 100 bp. The minimum identity of repeats was set at 90% (Hamming distance of 1). These repeat sequences were manually checked for alignment accuracy. Interspecific variation within the Echinostomatidae was calculated using MEGA v. 5.0 and MegAlign v. 5.01 [102,104].

### 2.4. Ribosomal Phylogenetic Analyses and Tree Reconstruction

To examine the phylogenetic and taxonomic position of the Echinostomatidae, three phylogenetic trees were reconstructed from the alignment of sequences of the 18S, ITS2, and 28S. We used rTU sequences of Echinostomatidae available in GenBank to create our phylogenetic datasets. *Paramphistomum cervi* Zeder, 1790 was included as an outgroup. Phylogenetic trees were reconstructed using Bayesian inference (BI), maximum likelihood (ML), and maximum parsimony (MP) methods. Bayesian inference was performed using the mixed model in MrBayes v. 3.1.1 and 1,000,000 metropolis-coupled Markov chain Monte Carlo generations. The first 250 trees were omitted as burn-in, and the remaining trees were used to calculate Bayesian posterior probabilities [105]. Maximum parsimony and ML analyses were performed using the Fitch criterion within PAUP v. 4.0 Beta 10 [106], and bootstrap support values were calculated in PAUP from 1000 bootstrap replicates with 10 random additions per replicate. Phylograms were viewed and drawn using FigTree V. 1.31 (http://tree.bio.ed.ac.uk/software/figtree/; accessed on 20 May 2025).

## 3. Results

### 3.1. Species Identifcation of E. miyagawai and P. bilobus

The 28S sequences obtained in this study for *E. miyagawai* (PV595723) and *P*. *bilobus* (PV569527) were aligned against the sequences of *E. miyagawai* and *P. bilobus* available in GenBank (KP065593, KT956945), showing sequence identities of 100% and 98.40%, respectively. Those two echinostomes were identified as *E. miyagawai* and *P. bilobus*, respectively.

### 3.2. Ribosomal Transcription Unit Features of the E. miyagawai and P. bilobus

In this study, the rTU sequences obtained were nearly complete for *E. miyagawai* (6893 bp) and *P. bilobus* (6790 bp), including the complete coding regions from 5′ terminus of the 18S rRNA gene to the 3′ terminus of the 28S rRNA gene. The positive bands of PCR products amplified were good, which were sequenced using the Sanger method (Figures S1 and S2). These sequences were submitted to GenBank with the accession numbers PV600287 and PV600288.

In rTU of *E. miyagawai*, the lengths of complete 18S, ITS1, 5.8S, ITS2, and 28S sequences were 1989 bp, 444 bp, 162 bp, 431 bp, and 3858 bp, respectively. The nucleotide composition of the 18S-28S, 18S, ITS1, 5.8S, and 28S sequences is biased toward C and G, with an overall C + G content of 51.98%, 50.68%, 52.93%, 53.09%, and 52.95%, respectively, and A + T content of 48.02%, 49.32%, 47.07%, 46.91%, and 47.05%, respectively. The nucleotide composition of ITS2 is biased towards A and T, with an overall A + T content of 50.35% and C + G content of 49.65%. A total of 52 repeat sequences were identified, including 11 forward, 27 reverse, 7 complement, and 7 palindromic repeats. The 28S sequence contained the most repeats, with a total of 41 repeat sequences (Table S2). The 18S sequence of *E. miyagawai* shows the highest sequence similarity with *E. miyagawai*, *E*. *paraensei*, and *E*. *revolutum*, reaching 99.20%. The ITS and 28S sequences of *E. miyagawai* show the highest sequence similarity with *E. miyagawai*, reaching 97.20% and 100%, respectively. The 18S, ITS, and 28S sequences of *E. miyagawai* show the lowest sequence similarity with *Mesorchis denticulatus* Rudolphi, 1802, *Euparyphium capitaneum* Rudolphi, 1802, and *P*. *bilobus*, reaching 95.70%, 80.20%, and 84.40%, respectively.

In the rTU of *P. bilobus*, the lengths of complete or nearly complete 18S, ITS1, 5.8S, ITS2, and 28S sequences were 1929 bp (nearly complete), 419 bp, 162 bp, 432 bp, and 3848 bp (nearly complete), respectively. The nucleotide composition of the 18S-28S, 18S, ITS1, 5.8S, and 28S sequences is biased toward C and G, with overall C + G content of 52.12%, 50.49%, 52.98%, 53.09%, and 53.07%, respectively, and A + T content of 47.88%, 49.51%, 47.02%, 46.91%, and 46.93%, respectively. The nucleotide composition of ITS2 nucleotide composition is biased towards A and T, with an overall A + T content of 50.23% and C + G content of 49.77%. A total of 55 repeat sequences were identified, including 11 forward, 25 reverse, 9 complement, and 10 palindromic repeats. The 28S sequence contained the most repeats, with a total of 45 repeat sequences (Table S3). The 18S sequence of *P*. *bilobus* shows the highest sequence similarity with *E*. *miyagawai* and *E*. *revolutum*, reaching 98.90%, and the lowest sequence similarity with *M*. *denticulatus* and *Euparyphium melis* Dietz 1909, reaching 95.90%. The ITS and 28S sequences of *P*. *bilobus* show the highest sequence similarity with *P*. *bilobus*, reaching 94.60% and 98.40%, respectively, and the lowest sequence similarity with *Pegosomum asperum* Rudolphi, 1802 and *Pegosomum saginatum* Rudolphi, 1802, reaching 81.60% and 77.60%, respectively.

### 3.3. Phylogenetic Analyses

**Figure 1:**
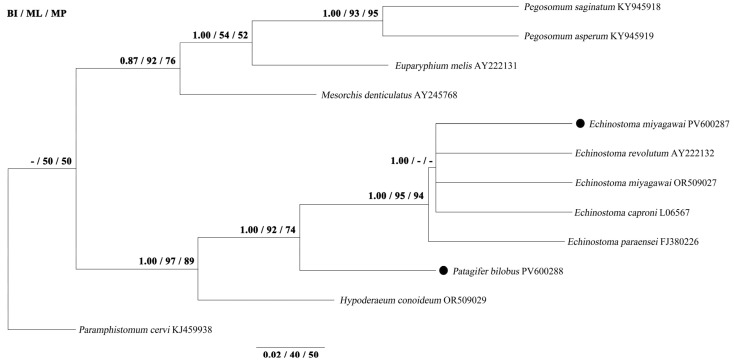
Genetic relationships of *E*. *miyagawai* and *P*. *bilobus* with other Echinostomatidae trematodes based on 18S sequence data. Phylogenetic analyses used Bayesian inference (BI), maximum likelihood (ML), and maximum parsimony (MP), with *P*. *cervi* as the outgroup. The scale bar indicates Posterior Probability. ● was the sequence obtained in this study.

**Figure 2:**
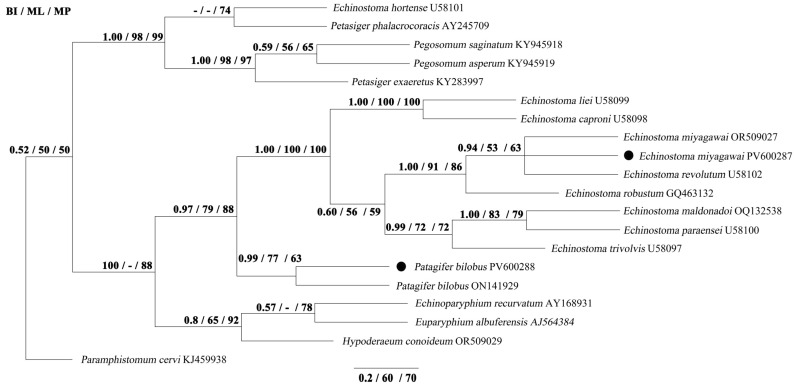
Genetic relationships of *E*. *miyagawai* and *P*. *bilobus* with other Echinostomatidae trematodes based on ITS sequence data. Phylogenetic analyses used Bayesian inference (BI), maximum likelihood (ML), and maximum parsimony (MP), with *P*. *cervi* as the outgroup. The scale bar indicates Posterior Probability. ● was the sequence obtained in this study.

**Figure 3:**
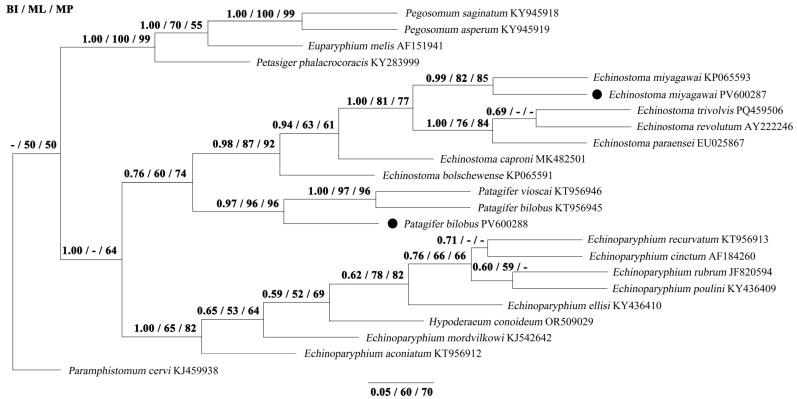
Genetic relationships of *E*. *miyagawai* and *P*. *bilobus* with other Echinostomatidae trematodes based on 28S sequence data. Phylogenetic analyses used Bayesian inference (BI), maximum likelihood (ML), and maximum parsimony (MP), with *P*. *cervi* as the outgroup. The scale bar indicates Posterior Probability. ● was the sequence obtained in this study.

Phylogenetic analyses using three methods (BI, ML, and MP) yielded identical tree topologies based on 18S, ITS, and 28S sequences, respectively (Figure 1, Figure 2 and Figure 3). The phylogenetic tree of 18S splits into two large clades. The first clade contains the species of *Pegosomum*, *Euparyphium*, and *M*. *denticulatus*. In the second clade, *E*. *miyagawai* from this study clusters with *E*. *miyagawai*, *E*. *revolutum*, and *E*. *caproni*, and *P*. *bilobus* formed a sister taxon with them. The phylogenetic tree of ITS splits into two large clades. The first clade contains the species of *Pegosomum*, *Petasiger*, and *E*. *hortense*. The second clade splits into two large groups. In the first group, *Echinostoma* species cluster together, and *E*. *miyagawai* from this study clusters with *E*. *miyagawai* and *E*. *revolutum*. *Patagifer bilobus* from this study clusters with *P*. *bilobus*, forming a sister taxon with *Echinostoma* species. All of the *Echinostoma* species cluster together, except *E*. *hortense*. The phylogenetic tree of 28S splits into two large clades. The first clade contains the species of *Pegosomum*, *Petasiger*, and *Euparyphium*. The second clade splits into two large groups. One group contains the species of *Echinostoma* and *Patagifer*, and the other contains the species of *Echinoparyphium* and *Hypoderaeum*. In the first group, *E*. *miyagawai* from this study clusters with *E*. *miyagawai*. *Patagifer bilobus* from this study clusters with *P*. *bilobus* and *P*. *vioscai*. In this study, the results for 28S were similar to those for the 18S and ITS sequences: *Echinostoma* species cluster together, and *Patagifer* species form a sister taxon with them. Further characterization of the phylogeny of *Echinostoma* and *Patagifer* species will need to wait until additional genomic trematode data have been deposited in GenBank.

## 4. Discussion

Echinostomes are intestinal parasites characterized by the “collar-spines”, a complex life cycle, and a broad range of definitive hosts that include humans. And Echinostomatidae are not only an overlooked food-borne pathogen but also a sensitive indicator of the complex ecological interactions among water bodies, snails, and hosts. Their control is of practical significance for public health, livestock production, and ecological security. *Echinostoma miyagawai* and *P*. *bilobus* both belong to Trematoda, Digenea, and Echinostomatidae. The taxonomic status of *E*. *miyagawai* has long been a focus of scholarly attention. *Echinostoma miyagawai* was first described by Ishii in 1932. And later, in 1937, Beaver [107] treated the species as synonym of *E*. *revolutum* according to morphological resemblance. But Bashkirova [108] and Skrjabin & Bashkirova [109] disagreed with their viewpoints and reinstated the two as distinct on both morphological and ecological grounds. In contrast, Kanev [110] dismissed *E*. *miyagawai* altogether, sinking it into *Echinostoma echinatum* Zeder, 1803. Kostadinova et al. [54,55] re-established *E*. *miyagawai* using distinctive cercarial chaetotaxy and detailed morphometrics of larvae and adults, clearly separating it from *E*. *revolutum*. Fried & Graczyk [111] and Toledo et al. [112] later concurred with this recognition. Finally, Faltýnková et al. [21] redescribed *E*. *miyagawai* adults from ducks across Central and Western Europe, placing *E*. *friedi* into synonymy with *E*. *miyagawai*. In addition, the sequences of *E*. *miyagawai* were obtained from both the definitive host and the intermediate host [57]. In conclusion, all available evidence supports the status of *E*. *miyagawai* as a valid species. Heneberg suggested that besides the issues with morphological species identification, it is important to highlight that further analyses should also refrain from raising conclusions that are based solely on variations in only a few informative sites, as the otherwise hypervariable genes, such as ITS1 or ITS2, are nearly invariable across the “revolutum” group complex [113]. In this study, the body of *E*. *miyagawai* is dorsoventrally flattened, robust and muscular, elongated and leaf-like, tapering slightly anteriorly and rounded posteriorly. The anterior end exhibits the characteristic echinostome features: an oral sucker and a head collar. The oral sucker is small, while the head collar is well-developed, prominent and muscular, armed with collar-spines numbering 37 in total, and the testes are petal-shaped. These characteristics are consistent with descriptions provided by other authors [50,51].

The genus *Patagifer* is a small genus of echinostomatids, which mainly parasitize birds of the family Threskiornithidae [88]. Besides Threskiornithidae birds, Patagifer species have also only been reported in birds of the families Ardeidae, Podicipedidae, and Scolopacidae [87,99]. This study reported for the first time a new host for the genus Patagifer, the *G*. *japonensis* (Gruidae), thereby enriching the host diversity of the genus Patagifer. The classification and species inventory of the genus Patagifer have been intricate and inconsistent, largely because the defining traits of the species exhibit significant phenotypic variability. In 1909, Dietz established the genus Patagifer, which includes *Distoma bilobum* Rudolphi, 1819 (syn. *P*. *bilobus*) and *Patagifer consimilis* Dietz, 1909. This was done to differentiate species characterized by a unique deep dorsal incision and a prominent ventral notch in the collar, features that are distinctive within the Echinostomatidae family and result in a characteristic bilobed appearance. Despite its relatively small size, the genus Patagifer has a complex taxonomic history and species composition. This complexity is largely due to the ambiguous description provided by Dietz (1910), which attributed significant variation to the type species *P*. *bilobus*. Additionally, the long-standing issue of insufficient or imprecise descriptions and poor differential diagnoses for newly recognized taxa has further complicated the synonymy. In 1956, Skrjabin & Bashkirova considered *P*. *bilobus*, *Patagifer consimilis* Dietz, 1909, *Patagifer parvispinosus* Yamaguti, 1933, and *Patagifer wesleyi* Verma, 1936 to be indistinguishable and questioned the distinct species status of *P*. *parvispinosus* and *P*. *wesleyi* [109]. In 1968, Machida considered that *Patagifer chandrapuri* Srivastava, 1952 and *Patagifer sarai* Saksena, 1957 were synonyms of *P*. *bilobus* [114]. In 1970, Jain & Srivastava considered that *Patagifer simerai* Nigam, 1944 was a synonym of *P*. *bilobus* [115]. In 1982, Srivastava considered that *P*. *simarai* and *P*. *sarai* were synonyms of *P*. *bilobus* [116]. Previous researchers have identified genus Patagifer based on their ecological characteristics, such as the ratio of the collar width to the body width, the proportion of the suckers, and the shape of the testes. However, there is no general consensus on the importance of these characteristics. Until 2008, Faltýnková et al. conducted the most comprehensive taxonomic study on the genus Patagifer and identified 11 species of Patagifer [87]. However, in the same year, Dronen & Blend did not follow the species identification key provided by Faltýnková et al. and described a 12th species, *Patagifer lamothei*, from the white ibis (*Eudocimus albus*) in the United States [99]. Faltýnková et al. considered that the characteristics of *P*. *bilobus* include the following: a ribbon-like body with almost parallel edges; a collar with a deep dorsal incision and a narrow ventral notch, bearing 52–58 collar-spines; and four subequal corner spines (one of which is slightly smaller) on each ventral lobe, which are smaller than the largest lateral spine. And *P*. *bilobus* in this study conformed to the morphological characteristics described above, and it had 54 collar-spines.

Qiqihar City is located in northeastern China, on the Songnen Plain, between 122°24′–126°41′ E and 46°13′–48°56′ N. It has a cold-temperate continental climate: winters are cold, summers are warm and humid, and the four seasons are distinct. The Nen River flows through the city, providing abundant water resources. The Nen River is approximately 1370 km long, playing an irreplaceable role in the local ecological environment and economic development. *Echinostoma miyagawai* was collected from domestic ducks in the Nen River basin in Qiqihar City. Domestic ducks in Northeast China originated from the wild mallard (Anas platyrhynchos) and were domesticated and selectively bred by local farmers over several centuries. Today, their populations are most numerous along the Songhua River basin and in the Songnen Plain. The Nen River basin is rich in aquatic animal resources, including a variety of fish and snails, which facilitates the transmission of the *E*. *miyagawai*. The kinds of fish in Nen River include the following: I. The family Cyprinidae: *Cyprinus carpio haematopterus* Temminck & Schlegel, 1842; *Ctenopharyngodon idellus* Cuvier & Valenciennes, 1844; *Leuciscus waleckii* Dybowski, 1869; *Hemiculter leucisculus* Basilewsky, 1855; *Xenocypris argentea* Günther, 1868; *Rhodeus sericeus* Pallas, 1776; and so on. II. The family Cobitidae: *Nemacheilus nudus* Blyth, 1860; *Misgurnus mohoity* Dybowski, 1869; *Paramisgurnus dabryanus* Sauvage, 1878. III. Others: *Siniperca chuatsi* Basilewsky, 1856; *Sander lucioperca* Linnaeus, 1758; *Pelteobagrus fulvidraco* Richardson, 1846; *Pelteobagrus nitidus* Sauvage & Dabry, 1874; *Lota lota* Linnaeus, 1758; *Pungitius sinensis* Guichenot, 1869. The kinds of snails in Nen River include the following: *Radix auricularia* Linnaeus, 1758; *Radix ovata* Draparnaud, 1805; *Radix plicatula* Benson, 1842; *Radix pereger* Müller, 1774; *Viviparus chui* Gray, 1847; *Cipangopa chinensis* Gray, 1834; *Bellamya aeruginosa* Reeve, 1863; *Semisulcospira amurensis* Gerstfeldt, 1859; Unio dougladiae Griffith & Pidgeon, 1834. The intermediate hosts of *E*. *miyagawai* that have been reported include *P. planorbis*, *A*. *vortex*, *L*. *truncatula*, *L*. *stagnalis*, and *L*. *palustris*, as shown in Table 1. However, the snail species reported above do not overlap with those in the Nen River, which implies that *E*. *miyagawai* may have a new intermediate host. This hypothesis will be tested in future studies. *Patagifer bilobus* was collected from *G. japonensis* in the Zhalong National Nature Reserve. The Zhalong National Nature Reserve is located in the western Songnen Plain of Heilongjiang Province, in the marshy reed wetlands of the lower Wuyur River basin. Its geographic coordinates are 123°47′–124°37′ E and 46°52′–47°32′ N, and the reserve covers a total area of 210,000 hectares. The Zhalong National Nature Reserve is a wetland ecosystem dominated by reed marshes, interspersed with lakes, meadows, grasslands, and saline–alkali lands. Zhalong is hailed as the “cradle of cranes”. Of the 15 crane species on Earth, 9 are found in China, and 6 of them are found in Zhalong: the Red-crowned Crane, Siberian (White) Crane, Hooded Crane, White-naped Crane, Common Crane, and Demoiselle Crane. Among these, the global population of Red-crowned Cranes is roughly 2000 individuals, and Zhalong alone hosts more than 400 breeding birds, making it the largest breeding ground for this species in the world. *Grus japonensi* lead a migratory life and transfer parasites between ecosystems during migration. The Zhalong Nature Reserve is not only a cornerstone of the global crane-conservation network but also a quintessential example of northern China’s wetland ecosystems, holding an irreplaceable strategic position in worldwide wetland and avian conservation. The kinds of fish in the Zhalong Nature Reserve include the following: *Carassius auratus* Linnaeus, 1758; *Cyprinus carpio* Linnaeus, 1758; *Ctenopharyngodon idella* Valenciennes, 1844; *Hypophthalmichthys molitrix* Valenciennes, 1844; *Aristichthys nobilis* Richardson, 1845; *Pseudorasbora parva* Temminck & Schlegel, 1846; Tachysurus fulvidraco; *Channa argus* Richardson, 1846; *Misgurnus anguillicaudatus* Cantor, 1842; and so on. The kinds of amphibians in the Zhalong Nature Reserve include the following: *Salamandrella keyserlingii* Dybowski, 1870; *Bufo gargarizans* Cantor, 1842; *Strauchbufo raddei* Kessler, 1878; *Hyla immaculata* Boettger, 1888; *Pelophylax nigromaculatus* Hallowell, 1861; *Rana amurensis* Boulenger, 1886. As for the latest publicly available information, a complete species list of gastropods (mollusca) in the Zhalong National Nature Reserve has not yet been published. The intermediate host of *P*. *bilobus* remains undetermined, and relevant information will be investigated in future studies.

In this study, the nearly complete rTU sequences were obtained for *E*. *miyagawai* (6893 bp) and *P*. *bilobus* (6790 bp). The nearly complete rTU sequence of *E*. *miyagawai* (OR509027) in the NCBI database was found in only one record, which was isolated from *Anas platyrhynchos* domesticus in Thailand. In rTU of *E*. *miyagawai* (OR509027), the lengths of complete 18S, ITS1, 5.8S, ITS2, and 28S sequence were 1988 bp, 417 bp, 160 bp, 428 bp, and 3861 bp, respectively. Compared with the sequence lengths of various regions of *E*. *miyagawai* in this study, discrepancies were observed between the two sequences. Alignment revealed several missing nucleotides, most notably a 26bp deletion within the ITS1 region. The 18S, ITS1, 5.8S, ITS2, and 28S sequence similarity of *E*. *miyagawai* (OR509027 and obtained this study) were 99.3%, 93.9%, 98.8%, 99.3%, and 99.8%, respectively. The 18S-28S, 18S, ITS1, 5.8S, and 28S sequence nucleotide composition of *E*. *miyagawai* are biased toward C and G, and the ITS2 nucleotide composition are biased toward A and T. The rTU of *E*. *miyagawai* had four types of repeat sequences, forward, reverse, complement, and palindromic repeats, and repeat sequences of 28S were the most. Internal repeats appear to be characteristic of the 28s evolution in different groups of organisms [91,117]. The sequences of *P*. *bilobus* in the NCBI database are limited. Currently, only partial ITS sequences, 28S sequences, and nad1 sequences are available. This study was the first time to amplify the nearly complete rTU sequences of *P*. *bilobus*. Not only does this fill the gap in the molecular sequence data of Chinese *Patagifer* spp., but it also reports a new host—*G. japonensis*. The ITS sequence similarity of *P*. *bilobus* (ON141929 and obtained this study) was 94.6%. The 28S sequence similarity of *P*. *bilobus* (ON141919 and obtained this study) was 98.4%. The 18S-28S, 18S, ITS1, 5.8S, and 28S sequence nucleotide composition of *P*. *bilobus* are biased toward C and G, and the ITS2 nucleotide composition are biased toward A and T. The bases (A, T, G, and C) are the fundamental components of DNA and RNA, and they play multiple important roles in the structure, function, and regulation of the genome. By encoding amino acids, regulating gene expression, participating in gene replication and recombination, and influencing gene function through modifications, the bases have played a key role in biological evolution and adaptation to the environment. In bioinformatics research, analyzing base sequences and their modifications can help us better understand the function and evolutionary mechanisms of the genome. And 28S sequence of *P*. *bilobus* also had the most repeat sequences, which is consistent with *E*. *miyagawai*. These repeat sequences have multiple functions in the genome, including gene regulation, gene replication, genome recombination, and gene silencing. They play an important role in the evolutionary process of organisms, influencing gene expression and genome structure to adapt to different environmental pressures. In bioinformatics research, the identification and analysis of these repeat sequences can help us better understand the function and evolutionary mechanisms of the genome.

Phylogenetic analyses using three methods (BI, ML, and MP) yielded identical tree topologies based on 18S, ITS, and 28S sequences, respectively. The results obtained from the three methods are similar. All Echinostoma species, except for *E*. *hortense*, cluster within a single clade, and Patagifer species form a sister clade with them. This is consistent with a previous study in which Echinostoma species cluster together, and Patagifer species formed a sister taxa with them, using 28S sequence with BI and ML methods [86]. Interestingly, all of the Echinostoma species cluster together, except *E*. *hortense*. This is consistent with a previous study in which *E*. *hortense* was not clustered together with Echinostoma species, while it clustered with Fasciola species, using mtDNA sequences [49,118]. And it is also consistent with a previous study using the 28S rRNA gene in which Echinostoma species cluster together on one branch, and *Isthmiophora hortensis* Lache, 1909 (Syn. *E*. *hortense*) is a sister taxa and cluster together with Petasiger species [86]. Interestingly, Echinostoma species in the phylogenetic tree all belong to 37 “collar-spines”, except *E*. *hortense*, and *E*. *hortense* has 27–28 “collar-spines” [34]. Similar ambiguous results regarding the location of *I*. *hortensis* based on both 28S and ITS2 sequence phylogenies have been inferred [119]. We speculate that the reason why *E*. *hortense* was not clustered together with other Echinostoma species might be due to the different numbers of collar-spines. The taxonomic status of *E*. *hortense* still needs further research to be explored. Further characterization of the species of Echinostoma and Patagifer phylogeny will need to wait until additional genomic trematode data has been deposited in GenBank. These findings not only provide new insights into the phylogenetic relationships of Echinostoma and Patagifer species, but they also highlight the importance of integrating multiple molecular markers and analytical methods in evolutionary studies. Future research should further explore the morphological and ecological characteristics of these species to better understand their evolutionary history and adaptive differentiation. Moreover, with the accumulation of more genomic data and advancements in technology, we expect to more comprehensively resolve the phylogenetic relationships of these species, providing a stronger scientific basis for biodiversity conservation and disease control.

## 5. Conclusions

In this study, we successfully obtained the nearly complete rTU sequences of *E*. *miyagawai* and *P*. *bilobus* and conducted detailed sequence and phylogenetic analyses. The acquisition of these sequence data has greatly enriched our understanding of the genetic characteristics of these two species of Echinostomatidae. The results of the phylogenetic analysis provide molecular evidence for the morphological classification of the genus Echinostoma and underscore the importance of integrating morphological features and molecular data in the taxonomic studies of trematodes. Additionally, the species of Patagifer form a sister clade with Echinostoma species, a result that further clarifies the phylogenetic position of Patagifer within the family Echinostomatidae and offers new insights into the evolutionary relationships within this family. It is worth noting that this study, for the first time, determined the nearly complete rTU sequence of *P*. *bilobus*. This achievement fills the gap in the molecular data of Patagifer and lays the foundation for future genetic research. Moreover, we have also reported for the first time a new host for *Patagifer* spp., *G*. *japonensis*. This discovery not only expands our knowledge of the host range of *Patagifer* spp. but also provides a new case for studying the interactions between trematodes and their hosts. From a phylogenetic perspective, more detailed genetic analyses will provide valuable information for the taxonomy, population genetics, and phylogenetics of the family Echinostomatidae. These data will serve as important molecular markers to help reveal the evolutionary history, population structure, and phylogenetic relationships among species within the family. Future research can further utilize these molecular markers, in combination with additional genomic data, to delve into the phylogenetic relationships of the Echinostomatidae and their adaptive evolution in different ecological environments. Moreover, these research findings will also provide a scientific basis for the prevention and control of trematode diseases, aiding in the development of more effective public health strategies.

## Figures and Tables

**Table 1:** List of related information *Echinostoma* spp. and *Patagifer* spp. available in PubMed database.

Species and Nominator	Collar-spines	Intermediate Host	Geographical Distribution	Definitive Host	Literature Sources
*Echinostoma bolschewense* (Kotova, 1939) Nasincova, 1991	37	*Viviparus contectus* Millet, 1813	Russia	*Mesocricetus auratus* Waterhouse, 1839; *Gallus gallus* Linnaeus, 1758	[8]
*Echinostoma caproni* Richard, 1964	37	*Biomphalaria* spp.; *Bulinus* spp.; *Helisoma duryi* Wetherby, 1879; *Lymnaea natalensis* Krauss, 1848; *Physa acuta* Draparnaud, 1805; *Planorbarius corneus* Linnaeus, 1758	Madagascar; Egypt; Kenya	Mice; hamsters; rats; chicks; pigeons; finches; *Crocidura olivieri* Lesson, 1827; *Falco newtoni* Gurney, 1863	[9,10,11]
*Echinostoma chankense* Besprozvannykh, 2001	27	*Anisus centrifugops* Prozorova & Starobogatov, 1997; *Helicorbis sujfunensis* Starobogatov, 1957; *Amuropaludina praerosa* Gerstfeldt, 1859; *Lymnaea auricularia* Linnaeus,1758	Russia	*Rattus norvegicus* Linnaeus, 1766	[6,12]
*Echinostoma cinetorchis* Ando & Ozaki, 1923	37	*Gyraulus convexiusculus* Macleay, 1873; *Hippeutis cantori* Benson, 1850; *Segmentina* spp., *Hemisphaerula* spp., *Radix auricularia coreana* Adams, 1866; *Austropeplea ollula* Gould, 1859 *Fossaria truncatula* Müller, 1774; *Corbicula fluminea* Müller, 1774;	Korea; China; Vietnam	Humans; dogs; ducks; *Rattus argentiventer* Robinson & Kloss, 1916; *Bandicota indica* Bechstein, 1800	[13,14,15,16,17,18,19]
*Echinostoma friedi* Toledo et al., 2000	37	*Lymnaea peregra* Müller, 1774 *Lymnaea* *corvus* Gmelin, 1791; *Gyraulus chinensis* Dunker, 1848; *Physella acuta* Draparnaud, 1805	Spain	Albino rats; golden hamsters; chickens; *R. norvegicus*	[20,21]
*Echinostoma hortense* Asada,1926	27–28	*Acanthogobius flavimanus* Temminck & Schlegel, 1845; *Misgurnus anguillicaudatus*, Cantor 1842; *Odontobutis interrupta*, Iwata & Jeon, 1985; *Misgurnus mizolepis* Nichols, 1925; *Moroco oxycephalus* Bleeker, 1865; *Coreoperca kawamebari* Temminck & Schlegel, 1843; *Squalidus coreanus* Berg, 1906	China; Korea; Japan	Dogs; humans; *R*. *norvegicus*; *Felis catus* Linnaeus, 1758; *Rattus rattus* Linnaeus, 1758	[22,23,24,25,26,27,28,29,30]
*Echinostoma ilocanum* Garrison, 1908	49–51	*G*. *convexiusculus*	Thailand; Cambodia	Rats; humans	[31,32,33]
*Echinostoma liei* Jeyarasasingam et al., 1972	37	*Biomphalaria glabrata* Orbigny, 1835; *Biomphalaria alexandrina* Ehrenberg, 1831	Egypt	Domestic chicks; hamsters; *M*. *auratus*	[34,35,36,37,38]
*Echinostoma macrorchis* Ando and Ozaki, 1923	40–45	*Cipangopaludina chinensis malleata* Reeve, 1863; *Filopaludina martensi* Martens, 1860; *Filopaludina doliaris* Gould, 1844; *Filopaludina sumatrensis polygramma* Martens, 1860; *Bithynia siamensis* Lea, 1856; *Bithynia pulchella* Adams, 1853; *Anentome helena* Busch, 1847	LAO; Korea; Thailand; Japan	Mice; rats; hamsters; *Mogera tokudae* Kuroda, 1940; *Mogera wogura* Temminck, 1844; *Apodemus speciosus* Temminck, 1835;	[39,40,41,42,43,44]
*Echinostoma maldonadoi* Kostadinova, 2000	33–39	*Stenophysa marmorata* Guilding, 1828	Brazil	*Meriones unguiculatus* Milne-Edwards, 1867	[45]
*Echinostoma mekongi* Cho et al., 2020	37	*F. martensi*; *A. helena;* *F*. *sumatrensis polygramma*	Cambodia; Thailand	Humans; *M*. *auratus*	[46,47,48]
*Echinostoma miyagawai* Ishii, 1932	37	*Planorbis planorbis* Linnaeus, 1758; *Anisus vortex* Linnaeus, 1758; *Lymnaea truncatula* Müller, 1774; *Lymnaea stagnalis* Linnaeus, 1758; *Lymnaea palustris Müller*, 1774	Japan; China; Korean; Czech; Thailand; Bulgaria; Poland; LAO; Indonesia	Pigeons, ducks, humans, *Aythya fuligula* Linnaeus, 1758; *Anas platyrhynchos* Linnaeus, 1758	[21,49,50,51,52,53,54,55,56]
*Echinostoma nasincovae* Georgieva et al., 2014	37	*P. corneus*	Czech; Russia; Ireland	*G. gallus*; *M. auratus;* *Anas platyrhyn* Linnaeus, 1758	[21,57,58,59]
*Echinostoma novaezealandense* Georgieva et al., 2017	37	-	New Zealand	*A*. *platyrhynchos;* *Cygnus atratus* Latham, 1790; *Branta canadensis* Linnaeus, 1758	[60]
*Echinostoma paraensei* Lie & Basch, 1967	37	*B. glabrata*; *P. acuta*	Brazil	Hamsters; mice; rats; *R. norvegicus;* *Nectomys squamipes Brants*, 1827	[45,61]
*Echinostoma paraulum* Dietz, 1909	37	*L*. *stagnalis*	Austria; Russia; Germany	*A*. *fuligula*	[21,57]
*Echinostoma pseudorobustum* Dietz, 1909	37	-	Brazil	*G*. *gallus*	[62]
*Echinostoma revolutum* (Froelich, 1802) Dietz, 1909	37	*Ampullaceana balthica* Linnaeus, 1758; *B. Siamensis*; *F. martensi*; C. *Bithynia funiculata* Leach, 1818; *Clea helena* Philippi, 1847; *Eyriesia eyriesi* Morelet, 1865; *F. doliaris*; *F. sumatrensis polygramma*; *Indoplanorbis exustus* Deshayes, 1833; *L*. *Auricularia*; *L. stagnalis;* *Lymnaea tomentosa* Pfeiffer, 1855 *Lymnaea elodes* Say, 1821; *Radix auricularia* Linnaeus, 1758; *Stagnicola palustris* Müller, 1774	Germany; LAO; Thailand; Korea; China; Czech; England; Poland; Scotland; Canada; Vietnam; America; Finland; Cambodia	Ducks; humans; rats; hamsters; *A*. *fuligula* *G*. *Gallus*; *B. canadensis;* *Grus japonensis* Müller, 1776;	[21,53,57,63,64,65,66,67,68,69,70,71,72]
*Echinostoma robustum* Yamaguti, 1935	37	*P*. *acuta*; *L*. *elodes*	China; Brazil; Bangladesh; Russia; America	Ducks; *G*. *gallus*; *A*. *platyrhynchos*	[73,74,75]
*Echinostoma trivolvis* Cort, 1914	37	*Lithobates sylvaticus* LeConte, 1825; *Physa gyrina* Say, 1821; *Helisoma trivolvis* Say, 1817; *Ladislavella elodes* Say, 1821; *Rana* spp. tadpoles	America	Mice; chicks; hamsters; *A*. *platyrhynchos*; *Ondatra zibethicus* Linnaeus, 1766	[76,77,78,79,80,81]
*Patagifer bilobus* (Rudolphi, 1819) Dietz, 1909	48–64	-	Mexico; Ukraine; America; Korea; Egypt; Argentina; Lithuania; China	*Plegadis chihi* Vieillot, 1817 *Nipponia nippon* Temminck, 1835; *Eudocimus albus* Linnaeus, 1758; *Bubulcus ibis* Linnaeus, 1758; *Plegadis falcinellus* Linnaeus, 1766; *Platalea minor* Temminck & Schlegel, 1849; *G. japonensis*	[82,83,84,85,86,87,88]
*Patagifer vioscai* Lumsden, 1962	53	*Pseudosuccinea columella* Say, 1817	America; South Africa	*E. albus*	[86,87,89]

**Table 2:** Higher taxa of *Echinostoma* spp. and *Patagifer* spp.

Biological Classification	*Echinostoma* spp.	*Patagifer* spp.
Phylum	Platyhelminthes	Platyhelminthes
Class	Trematoda	Trematoda
Subclass	Digenea	Digenea
Order	Plagiorchiida	Plagiorchiida
Suborder	Echinostomata	Echinostomata
Superfamily	Echinostomatoidea	Echinostomatoidea
Family	Echinostomatidae	Echinostomatidae
Gens	Echinostoma	Patagifer

## Data Availability

No new data were created or analyzed in this study.

## Supplementary Materials

The following supporting information can be downloaded at: https://www.mdpi.com/article/10.3390/biology14081101/s1, Figure S1: Agarose gel electropheoresis of rTU PCR products of *E*. *miyagawai* samples; Figure S2: Agarose gel electropheoresis of rTU PCR products of *P*. *bilobus* samples; Table S1: Sequences of primers used to amplify PCR fragments of 18S-28S rDNA of *Echinostoma miyagawa* and *Patagifer bilobus*; Table S2: Information of repeat sequences in the 18S-28S rDNA of *Echinostoma miyagawa*; Table S3: Information of repeat sequences in the 18S-28S rDNA of *Patagifer bilobus*.
