# Supplementary material for: The Nuclear Ribosomal Transcription Units of Two Echinostomes and Their Taxonomic Implications for the Family Echinostomatidae
Source: Biology (Basel). 2025 Aug 21;14(8):1101. doi: 10.3390/biology14081101 (PMC12383542; doi:10.3390/biology14081101)
Supplement: Supplementary file 1 [file biology-14-01101-s001.zip › Table S1.pdf]

Table S1. Sequences of primers used to amplify PCR fragments of 18S-28S rDNA of *Echinostoma miyagawa* and *Patagifer bilobus*.

Primer name	Primer sequence (5′-3′)	Amplified region	Size (kb)
18SF	GGCTCATTAAATCAGCTATGGTT	18S	~1.9
18SR	ACGACTTTTACTTCCTCTAAAT		
18S-28SF	ACAATGACGGTTTCAGCGAGTTT	18S-28S	~1.4
18S-28SR	CACAAACAACCCGACTCCAAGG		
28S1F	TAGGCAATGTGGTGTTTAGGT	28S	~1.2
28S1R	GCGGTCCTCCACCAGAGTTTC		
28S2F	CAATAGTCTGTGGTGTAGTGG	28S	~2.2
28S2R	AATAAGCAAAGAAACAATGAC		
28S3F	CGTTCAGTCCTAATCCCTC	28S	~1.0
28S3R	GTAGCCAAATGCCTCGTC		
