# Supplementary material for: The Nuclear Ribosomal Transcription Units of Two Echinostomes and Their Taxonomic Implications for the Family Echinostomatidae
Source: Biology (Basel). 2025 Aug 21;14(8):1101. doi: 10.3390/biology14081101 (PMC12383542; doi:10.3390/biology14081101)
Supplement: Supplementary file 1 [file biology-14-01101-s001.zip › Table S2.pdf]

Table S2. Information of repeat sequences in the 18S-28S rDNA of *Echinostoma miyagawa*.

ID	Repeat Start 1	Type	Size (bp)	Repeat Start 2	Repeat distance	Gene
1	171	Complement	11	496	0	18S
2	185	Reverse	11	185	0	18S
3	242	Reverse	11	1487	0	18S
4	4	Reverse	12	1113	0	18S
5	1370	Reverse	12	1370	0	18S
6	949	Palindromic	12	949	0	18S
7	40	Reverse	10	185	0	ITS1
8	384	Reverse	10	384	0	ITS1
9	43	Palindromic	10	43	0	ITS2
10	333	Reverse	12	333	0	ITS2
11	200	Forward	23	274	0	ITS2
12	97	Forward	10	3512	0	28S
13	145	Forward	10	1778	0	28S
14	461	Forward	10	1760	0	28S
15	801	Forward	10	3091	0	28S
16	1260	Forward	10	2559	0	28S
17	1408	Forward	10	2002	0	28S
18	1484	Forward	10	3021	0	28S
19	1549	Forward	10	2711	0	28S
20	502	Forward	11	3213	0	28S
21	2139	Forward	11	3235	0	28S
22	147	Reverse	10	2299	0	28S
23	261	Reverse	10	2774	0	28S
24	389	Reverse	10	389	0	28S
25	990	Reverse	10	3753	0	28S
26	1057	Reverse	10	1057	0	28S
27	2146	Reverse	10	3305	0	28S
28	2613	Reverse	10	2613	0	28S
29	3086	Reverse	10	3086	0	28S
30	3144	Reverse	10	3391	0	28S
31	3235	Reverse	10	3235	0	28S
32	73	Reverse	11	73	0	28S
33	969	Reverse	11	2798	0	28S
34	1029	Reverse	11	1029	0	28S
35	2138	Reverse	11	3235	0	28S
36	2219	Reverse	11	3122	0	28S
37	225	Reverse	12	3069	0	28S
38	1022	Reverse	12	2754	0	28S
39	2138	Reverse	12	2138	0	28S
40	812	Reverse	13	812	0	28S
41	2678	Reverse	14	2678	0	28S

42	23	Complement	10	2609	0	28S
43	56	Complement	10	2217	0	28S
44	707	Complement	10	1707	0	28S
45	1130	Complement	10	1152	0	28S
46	1461	Complement	10	3478	0	28S
47	110	Complement	13	3231	0	28S
48	397	Palindromic	10	397	0	28S
49	548	Palindromic	10	1034	0	28S
50	654	Palindromic	10	1636	0	28S
51	3548	Palindromic	12	3548	0	28S
52	2030	Palindromic	13	3287	0	28S
