# Supplementary material for: The Nuclear Ribosomal Transcription Units of Two Echinostomes and Their Taxonomic Implications for the Family Echinostomatidae
Source: Biology (Basel). 2025 Aug 21;14(8):1101. doi: 10.3390/biology14081101 (PMC12383542; doi:10.3390/biology14081101)
Supplement: Supplementary file 1 [file biology-14-01101-s001.zip › Table S3.pdf]

Table S3. Information of repeat sequences in the 18S-28S rDNA of *Patagifer bilobus*.

ID	Repeat Start 1	Type	Size (bp)	Repeat Start 2	Repeat distance	Gene
1	12	Forward	11	1592	0	18S
2	130	Reverse	11	130	0	18S
3	1317	Reverse	12	1317	0	18S
4	116	Complement	11	441	0	18S
5	896	Palindromic	12	896	0	18S
6	273	Reverse	10	273	0	ITS1
7	387	Reverse	10	387	0	ITS1
8	214	Forward	11	288	0	ITS2
9	42	Palindromic	10	42	0	ITS2
10	365	Palindromic	10	365	0	ITS2
11	97	Forward	10	3510	0	28S
12	1405	Forward	10	2001	0	28S
13	1481	Forward	10	3019	0	28S
14	1546	Forward	10	2709	0	28S
15	1751	Forward	10	3114	0	28S
16	2407	Forward	10	3779	0	28S
17	501	Forward	11	3211	0	28S
18	894	Forward	11	2685	0	28S
19	2138	Forward	11	3233	0	28S
20	147	Reverse	10	2298	0	28S
21	227	Reverse	10	3067	0	28S
22	388	Reverse	10	388	0	28S
23	1056	Reverse	10	1056	0	28S
24	1107	Reverse	10	1331	0	28S
25	1580	Reverse	10	1810	0	28S
26	1778	Reverse	10	1778	0	28S
27	2145	Reverse	10	3303	0	28S
28	2611	Reverse	10	2611	0	28S
29	3142	Reverse	10	3389	0	28S
30	3233	Reverse	10	3233	0	28S
31	73	Reverse	11	73	0	28S
32	968	Reverse	11	2796	0	28S
33	1028	Reverse	11	1028	0	28S
34	2137	Reverse	11	3233	0	28S
35	2218	Reverse	11	3120	0	28S
36	1021	Reverse	12	2752	0	28S
37	2137	Reverse	12	2137	0	28S
38	811	Reverse	13	811	0	28S
39	898	Reverse	13	898	0	28S
40	2676	Reverse	14	2676	0	28S
41	23	Complement	10	2607	0	28S

42	56	Complement	10	2216	0	28S
43	706	Complement	10	1704	0	28S
44	1458	Complement	10	3476	0	28S
45	303	Complement	11	3650	0	28S
46	306	Complement	11	1174	0	28S
47	2570	Complement	11	2883	0	28S
48	110	Complement	13	3229	0	28S
49	358	Palindromic	10	2582	0	28S
50	396	Palindromic	10	396	0	28S
51	547	Palindromic	10	1033	0	28S
52	653	Palindromic	10	1633	0	28S
53	3806	Palindromic	10	3806	0	28S
54	3546	Palindromic	12	3546	0	28S
55	2029	Palindromic	13	3285	0	28S
