# Supplementary figures and images for: The Nuclear Ribosomal Transcription Units of Two Echinostomes and Their Taxonomic Implications for the Family Echinostomatidae
Source: Biology (Basel). 2025 Aug 21;14(8):1101. doi: 10.3390/biology14081101 (PMC12383542; doi:10.3390/biology14081101)

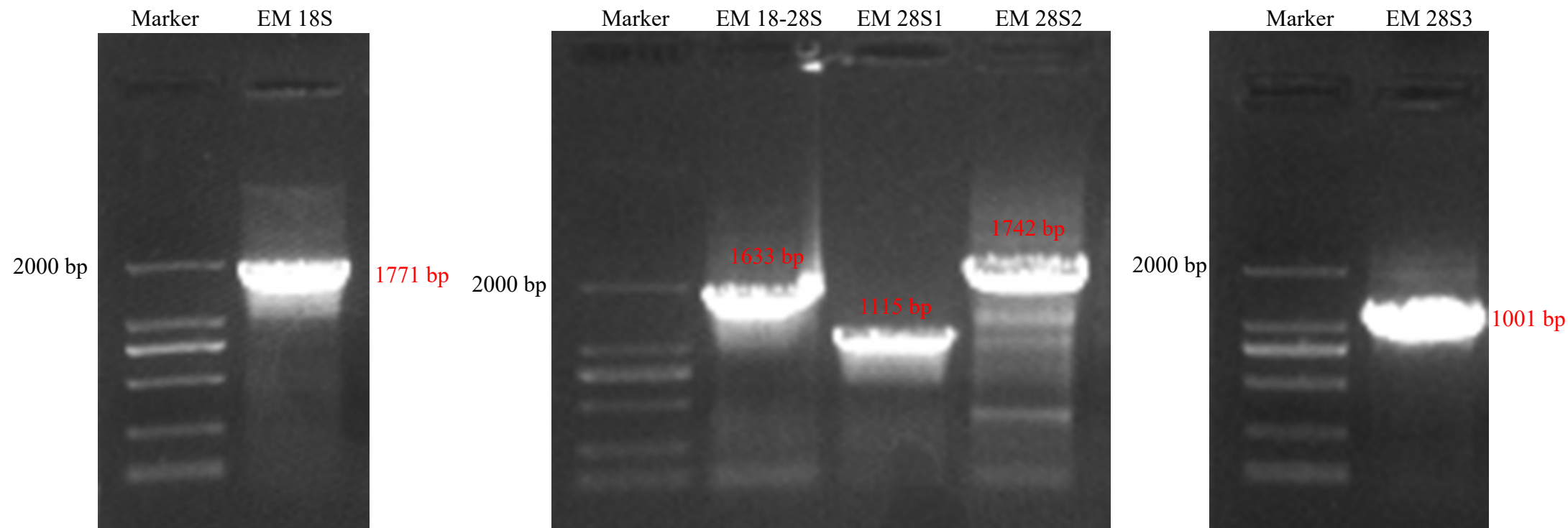

Fig. S1. Agarose gel electrophoresis of rTU PCR products of *E. miyagawai* samples

Supplement: Supplementary file 1 [file biology-14-01101-s001.zip › Figure S1.pdf]

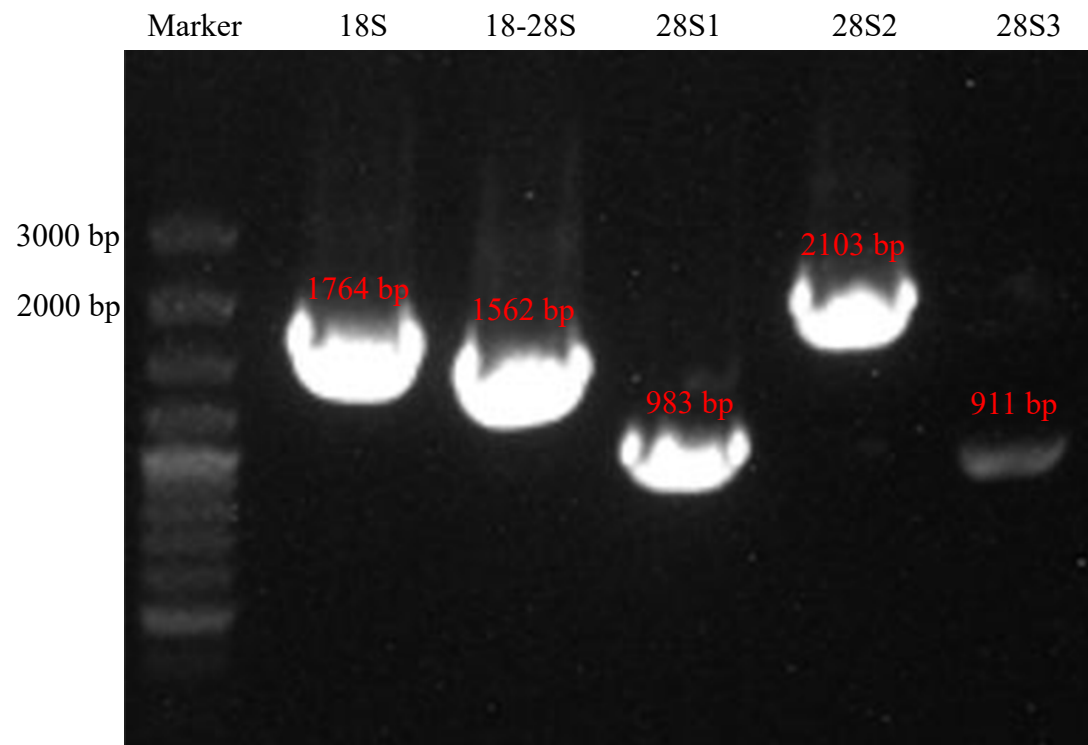

Fig. S2. Agarose gel electrophoresis of rTU PCR products of *P. bilobus* samples

Supplement: Supplementary file 1 [file biology-14-01101-s001.zip › Figure S2.pdf]
